# Protein-Rich Fraction of *Cnidoscolus urens* (L.) Arthur Leaves: Enzymatic Characterization and Procoagulant and Fibrinogenolytic Activities

**DOI:** 10.3390/molecules19033552

**Published:** 2014-03-21

**Authors:** Yamara A. S. de Menezes, Juliana Félix-Silva, Arnóbio A. da Silva-Júnior, Ivanise M. M. Rebecchi, Adeliana S. de Oliveira, Adriana F. Uchoa, Matheus de F. Fernandes-Pedrosa

**Affiliations:** 1 Laboratório de Tecnologia e Biotecnologia Farmacêutica, Departamento de Farmácia, Universidade Federal do Rio Grande do Norte, Rua Gal. Gustavo Cordeiro de Farias, s/n–Petrópolis, Natal, RN 59012-570, Brazil; E-Mails: yamaramenezes@yahoo.com.br (Y.A.S.M.); julianafelix_rn@hotmail.com (J.F.-S.); arnobiosilva@ufrnet.br (A.A.S.-J.); 2 Laboratório de Hematologia Clínica, Departamento de Análises Clínicas e Toxicológicas, Universidade Federal do Rio Grande do Norte, Rua Gal. Gustavo Cordeiro de Farias, s/n–Petrópolis, Natal, RN 59012-570, Brazil; E-Mail: ivaniserebecchi@gmail.com; 3 Departamento de Bioquímica, Universidade Federal do Rio Grande do Norte: Instituto de Medicina Tropical do Rio Grande do Norte, Campus Universitário, s/n–Lagoa Nova, Natal, RN 59078-970, Brazil; E-Mail: cisteana@yahoo.com.br; 4 Departamento de Biologia Celular e Genética, Universidade Federal do Rio Grande do Norte: Instituto de Medicina Tropical do Rio Grande do Norte, Campus Universitário, s/n–Lagoa Nova, Natal, RN 59078-970, Brazil; E-Mail: afuchoa@ufrnet.br

**Keywords:** *Cnidoscolus urens*, Euphorbiaceae, cysteine proteases, enzymatic characterization, fibrin(ogen)olytic, procoagulant

## Abstract

Proteolytic enzymes are important macromolecules in the regulation of biochemical processes in living organisms. Additionally, these versatile biomolecules have numerous applications in the industrial segment. In this study we have characterized a protein-rich fraction of *Cnidoscolus urens* (L.) Arthur leaves, rich in proteolytic enzymes, and evaluated its effects on the coagulation cascade. Three protein-rich fractions were obtained from the crude extract of *C. urens* leaves by precipitation with acetone. Fraction F1.0 showed higher proteolytic activity upon azocasein, and thus, was chosen for subsequent tests. The proteolytic activity of F1.0 on fibrinogen was dose-dependent and time-dependent. The extract demonstrated procoagulant activity on citrated plasma and reduced the APTT, not exerting effects on PT. Despite the fibrin(ogen)olytic activity, F1.0 showed no defibrinogenating activity *in vivo*. The fraction F1.0 did not express hemorrhagic nor hemolytic activities. The proteolytic activity was inhibited by E-64, EDTA and in the presence of metal ions, and increased when pretreated with reducing agents, suggesting that the observed activity was mostly due to cysteine proteases. Several bands with proteolytic activity were detected by zymography with gelatin, albumin and fibrinogen. The optimal enzymatic activity was observed in temperature of 60 °C and pH 5.0, demonstrating the presence of acidic proteases. In conclusion, these results could provide basis for the pharmacological application of *C. urens* proteases as a new source of bioactive molecules to treat bleeding and thrombotic disorders.

## 1. Introduction

Proteases (EC 3.4), also known as proteolytic enzymes or peptidases, are a large group of enzymes which play a significant role in many biochemical mechanisms to maintain metabolic processes of all organisms [1]. They have been isolated in recent years from plants (latex, fruits, and seeds) and animals (bee, snake, scorpion and spider venoms) [2]. Proteases may be used for pharmacological purposes, such as in digestive processes, blood coagulation and fibrinolysis. Proteases that affect the coagulation and fibrinolysis were isolated and characterized in plants, with reports on species of the families Amaryllidaceae, Apocynaceae, Euphorbiaceae, Moraceae and Moringaceae. Several plant enzymes belonging to the classes of serine proteases and cysteine proteases were observed to be active on the cascade of clotting factors [1,2,3,4,5,6,7,8,9,10,11].

Examples of serine proteases isolated from plants are ATFE (*Allium tuberosum*) [3,4], Hirtin (*Euphorbia hirta*) [12], LGP (*Synadenium grantii*) [6], AMP48 (*Artocarpus heterophyllus*) [1]; among cysteine proteases are ATFEII (*Allium tuberosum*) [3], “Pergularain e I” (*Pergularia extensa*) [11], Eumiliin (*Euphorbia milii var. hislopii*) [5]. All these proteases presented fibrinogenolytic activity. Ficin, a cysteine protease derived from *Ficus carica*, activated human Factor X and induced blood coagulation [9].

*Cnidoscolus urens* (L.) Arthur is a vegetal species belonging to family Euphorbiaceae that is widely distributed in Brazil. In folk medicine, it is used in treatment of cancer, hemorrhage, inflammation, pain, among other uses [13,14,15,16]. However, until this moment, there is no report regarding the isolation or characterization of proteases of this species with pharmacological applications.

In this study, we report for the first time the pharmacological properties of a protein-rich fraction of *C. urens* leaves, rich in proteolytic enzymes, evaluating its action on blood coagulation, more specifically its fibrin(ogen)olytic and procoagulant activities, suggesting significant therapeutic applications.

## 2. Results and Discussion

### 2.1. Azocaseinolytic Activity

Proteases are proteolytic enzymes naturally found in all organisms [17]. The interest in proteolytic enzymes has grown and shown great importance due to the variety of physiological activities that they play, in addition to their application in various industrial segments, including the pharmaceutical industry [2,7]. Proteases are involved in processes such as protein catabolism, blood clotting, cell growth and migration, tissue formation, morphogenesis in development, inflammation, tumor growth, activation of zymogens, release of peptide hormones and pharmacologically active proteins and also in precursor protein transport across membranes [18].

**Figure 1:**
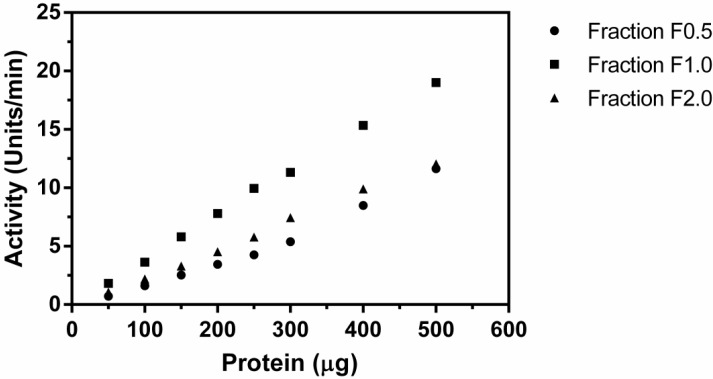
Azocaseinolytic activity of *C. urens* fractions F0.5, F1.0 and F2.0*.* Reaction mixture (350 µL) contained 100 µL of azocasein (1%) in 0.05 M Tris-HCl, 0.15 M NaCl, pH 7.5 incubated with different concentrations of *C. urens* fractions ranging from 50–500 µg for 30 min at 37 °C. Values represent mean ± SEM (*n =* 3).

In order to assess the presence of proteolytic activity in protein extracts of *Cnidoscolus urens*, an assay for azocaseinolytic activity was performed. Protein fractions (F0.5, F1.0 and F2.0) of *C. urens* leaves were obtained after precipitation of the crude extract at various concentrations of cold acetone (1:2, 1:1 and 2:1, v/v, acetone:extract). All fractions were submitted to proteolytic assay with azocasein (1%) as substrate. All fractions of *C. urens* hydrolyzed azocasein in a protein concentration dependent manner (Figure 1). Fraction F1.0 was the most active (*p <* 0.001 compared to F0.5 and F2.0) being therefore chosen to proceed with the other tests.

### 2.2. Eletrophoretic Profile and Zymography

**Figure 2:**
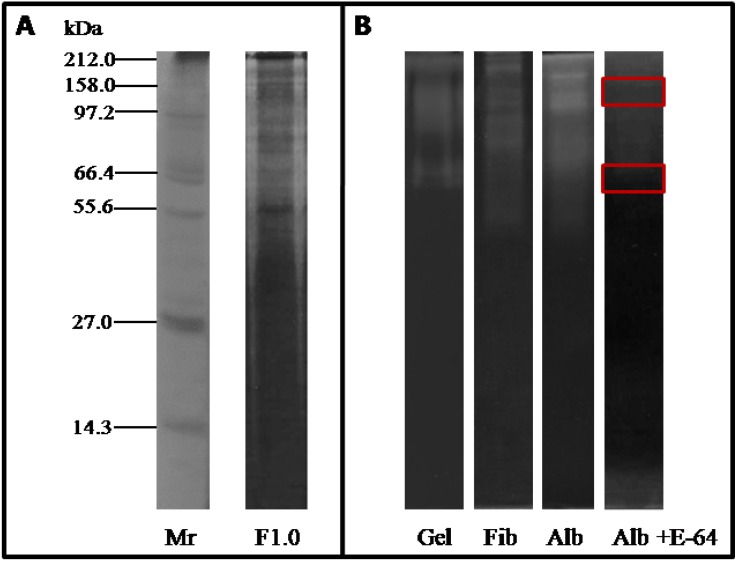
SDS-PAGE profile of fraction F1.0 proteins and in-gel protease assay (zymography). (**A**) Electrophoretic analysis in polyacrylamide gel (15%) of fraction F1.0 of *C. urens*. Lane Mr: molecular weight marker; Lane F1.0: fraction F1.0 of *C. urens* treated in non-reducing buffer. The gels were stained with silver staining; (**B**) Zymogram gels. To assess the proteolytic activity by zymogram technique, solution of 15% polyacrylamide was copolymerized with different substrates. After polymerization, the fraction F1.0 was applied to the gels at a concentration of 1.5 µg/µL, and the electrophoretic run was developed. Lane Gel: copolymerized gelatin; Lane Fib: copolymerized fibrinogen; Lane Alb: copolymerized albumin. Lane Alb + E-64: Inhibition of F1.0 at concentration of 1.5 µg/µL by E-64 1 mM in zymogram with albumin co-polymerized. The gels were stained with Coomassie brilliant blue R-250.

F1.0 was resolved into several protein bands ranging from 150 kDa to 6.5 kDa when subjected to SDS-PAGE (Figure 2A). The presence of bands with proteolytic activity upon gelatin, albumin and fibrinogen were detected by gel zymography, with molecular weights ranging from 150 kDa to 50 kDa, as observed in Figure 2B. Two of those bands (116.7 and 58.5 kDa) were not inhibited by E-64 when tested upon albumin. The present study shows that leaves of *C. urens* are an abundant source of proteolytic enzymes. Further inhibition assays employing specific protease inhibitors (E-64, PMSF and EDTA) and β-mercaptoethanol (reducing agent), suggested that the main proteases extracted from *C. urens* are cysteine proteases (data not shown).

### 2.3. Fibrinogenolytic Activity

**Figure 3:**
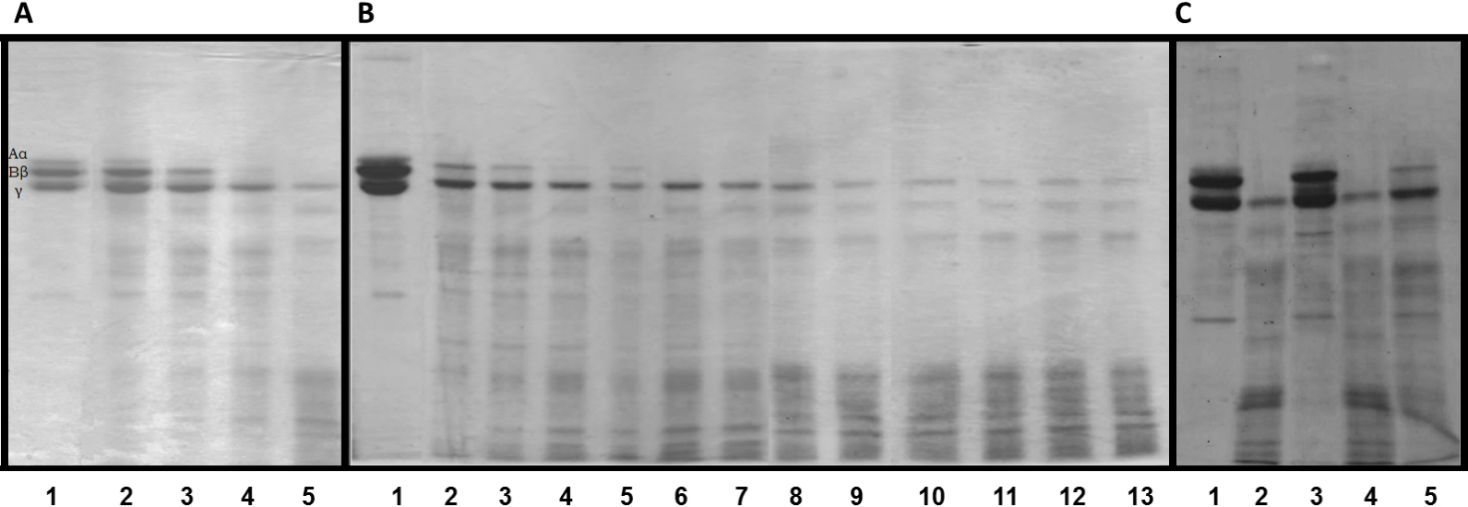
Fibrinogenolytic activity of *C. urens* fraction F1.0 analyzed on 12% SDS-PAGE under reducing conditions. (**A**) Evaluation of the dose-dependence of hydrolysis of fibrinogen by proteins fraction F1.0. Lane 1: pure fibrinogen (50 µg); Lane 2: fibrinogen + 0.02 µg/µL; Lane 3: fibrinogen + 0.04 µg/µL; Lane 4: fibrinogen + 0.09 µg/µL; Lane 5: fibrinogen + 0.18 µg/µL. The fraction F1.0 and fibrinogen were incubated at 37 °C for 1 h; (**B**) Kinetics of fibrinogen hydrolysis by fraction F1.0. Lane 1: pure fibrinogen (50 µg) incubated for 120 min without *C. urens* fraction F1.0, Lanes 2–11: fibrinogen (50 µg) incubated for with *C. urens* fraction F1.0 (0.18 µg/µL) at 37 °C for 30 sec, 1, 2, 3, 4, 5, 10, 15, 20, 30, 60 and 120 min in 0.05 M Tris-HCl, 0.15 M NaCl, pH 7.5 buffer; (**C**) Evaluation of the effect of specific inhibitors on the hydrolysis of fibrinogen. Lane 1: pure fibrinogen (50 µg); Lane 2: fibrinogen + fraction F1.0 *C. urens* protein (0.18 µg/µL); Lanes 3–5: fraction F1.0 of *C. urens* (0.18 µg/µL) pre-incubated with different inhibitors for 15 min (1 mM E-64, 1 mM PMSF and 1 mM EDTA, respectively), followed by incubation with 50 μg of bovine fibrinogen 37 °C for 1 h. The gels were stained with Coomassie brilliant blue R-250.

Among proteolytic enzymes, those which hydrolyze fibrinogen have been related to important effects on the coagulation cascade. Studies conducted with these molecules could contribute to the development of molecules of interest in the treatment of disorders associated with the coagulation cascade, beyond the possible application in the clinical laboratory [2]. The results obtained in this study clearly indicate the presence of fibrinogenolytic enzymes in *C. urens* leaves. The fibrinogen cleavage pattern was analyzed by SDS-PAGE under reducing conditions, evaluating dose- and time-dependence as well as inhibition studies with specific inhibitors to determine the class of active proteases (Figure 3). F1.0 preferentially hydrolyzed Aα and Bβ chains of fibrinogen. At the highest protein concentration tested (0.18 µg/µL), F1.0 completely hydrolyzed Aα and Bβ chains and partially degraded the γ chain (Figure 3A). Hydrolysis of Aα and Bβ chains of fibrinogen by F1.0 resulted in the appearance of low molecular weight fragments.

The kinetics of fibrinogen degradation was determined by incubation of enzyme source plus substrate for 0.5, 1, 2, 3, 4, 5, 10, 15, 20, 30, 60 and 120 min. The incubation of F1.0 with bovine fibrinogen showed that the enzyme hydrolyzed Aα chain very efficiently within 1 min of incubation, Bβ within 4 min, while the hydrolysis γ chain is slower (Figure 3B). The intensity of the hydrolyzed products increased over the incubation time. The γ chain was not completely hydrolyzed even after 120 min incubation at 0.18 µg/µL of protein. These data suggest that the susceptibility of fibrinogen to proteolytic digestion by fraction F1.0 was different for each chain (Aα > Bβ > γ).

In order to explore the class of protease involved, an inhibition study was carried out using different class-specific inhibitors (E-64, PMSF and EDTA). The fibrinogenolytic activity of F1.0 was inhibited by E-64 (Figure 3C), a cysteine protease inhibitor, but only partially inhibited by EDTA, whereas PMSF showed no inhibitory effect. These results suggest that F1.0 contains predominantly cysteine proteases. This is an interesting result since only few cysteine proteases from plant sources affecting hemostasis were characterized until this date.

### 2.4. Fibrinolytic Activity

Fibrinolysis is another important proteolytic event that is associated with wound healing. During physiological processes, plasmin is the key enzyme that hydrolyzes fibrin clots [19]. In the fibrinogenolytic activity assay, it was found that fibrinogen γ chain was cleaved at high protein concentration or an extended incubation time. It has been reported that, in general, proteases which hydrolyze fibrinogen γ subunit are also able to hydrolyze fibrin clot [7]. In view of this, the fibrinolytic activity of F1.0 was investigated.

**Figure 4:**
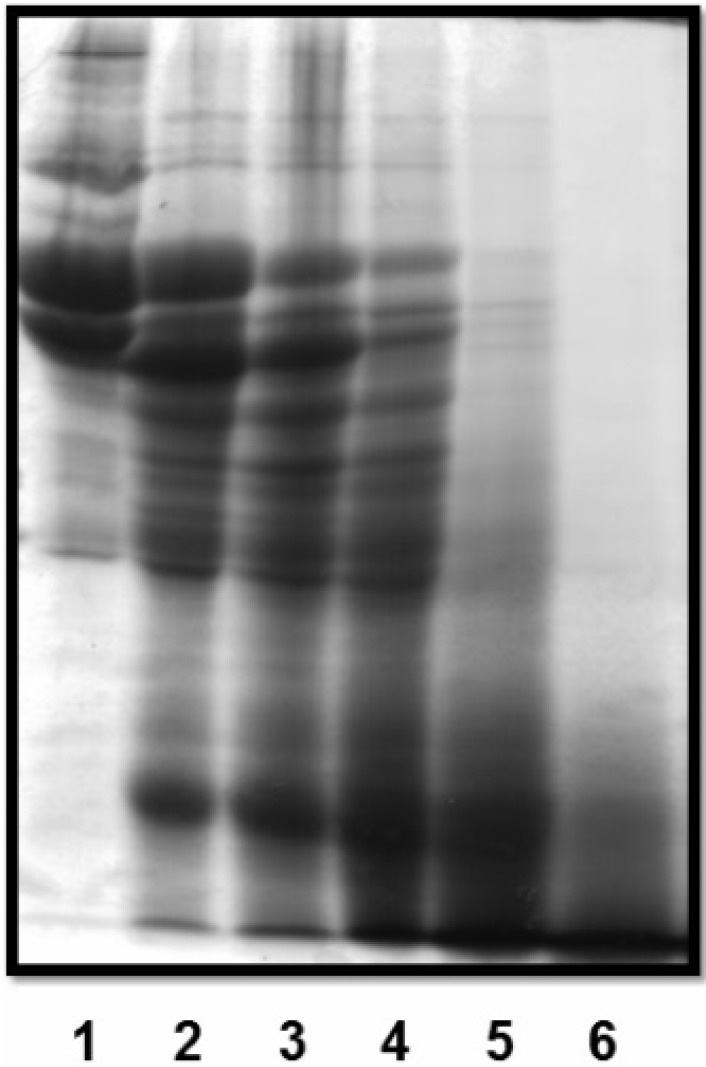
Dose-dependent hydrolysis of plasma clot by fraction F1.0 of *C. urens*. Washed plasma clot was incubated with various concentrations of fraction F1.0 in 0.05 M Tris-HCl, 0.15 M NaCl, pH 7.5 for 1 h at 37 °C. Lane 1: control (plasma clot alone); Lane 2: plasma clot + 0.09 µg/µL; Lane 3: plasma clot + 0.18 µg/µL; Lane 4: plasma clot + 0.37 µg/µL; Lane 5: plasma clot + 0.75 µg/µL; Lane 6: plasma clot + 1.5 µg/µL of fraction F1.0. The gel was stained with Coomassie brilliant blue R-250.

In order to verify the presence of fibrinolytic activity, plasma clots were incubated with F1.0 at different protein concentrations for 1 h at 37 °C. The reaction was stopped by addition of SDS-PAGE sample buffer in the presence of β-mercaptoethanol and immediately subjected to 100 °C for 5 min. The hydrolysis pattern was visualized by SDS-PAGE (Figure 4). We found that with increasing protein concentration, bands related to the fibrin clot are gradually hydrolyzed, confirming the presence of fibrinolytic activity. The protein concentration at which we observe the complete hydrolysis of the bands related to the fibrin clot is 1.5 µg/µL, a relatively high concentration when compared to those checked for fibrinogenolytic activity (0.18 µg/µL). This result suggests *C. urens* proteases have higher affinity to fibrinogen than to fibrin. Since plasma clots were used in this study, one possible reason for this result is the presence of plasmatic inhibitors and factor XIIIa (fibrin stabilizer factor), which could decrease the affinity of F1.0 to fibrin.

### 2.5. Procoagulant Activity

**Figure 5:**
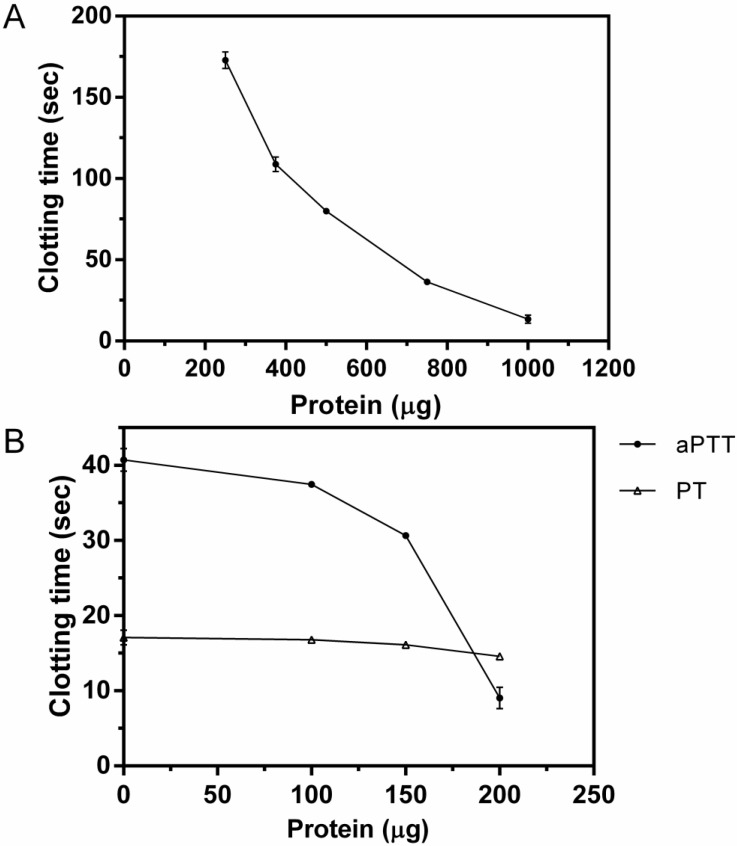
Effect of *C. urens* fraction F1.0 on clotting time of human plasma. (A) Detection of coagulant activity. The fraction F1.0 of *C. urens* (ranging from 250–1000 µg) was incubated with 150 µL of human plasma and the time for clot formation was recorded. (B) Effect of *C. urens* fraction F1.0 on the activated partial thromboplastin time (APTT) and prothrombin time (PT). *C. urens* fraction F1.0 proteins ranging from 100–200 µg were preincubated with 60 µL of human plasma for 1 min at 37 °C and the time for clot formation was recorded. The values represent mean ± SEM (*n =* 3).

The procoagulant activity of F1.0 was determined by measuring its effect on the clotting time of human citrated plasma in a digital coagulometer. The clotting time was reduced after addition of increasing concentrations of F1.0 (Figure 5A), indicating its dose-dependent procoagulant action. The minimum coagulant concentration, or that necessary to promote the coagulation of citrated plasma in 60 s, was equal to 653.81 µg of protein.

### 2.6. APTT and PT Tests

We also investigated the effect of F1.0 on blood coagulation by determination of activated partial thromboplastin time (APTT) and prothrombin time (PT). APTT decreased from 41 s to 14 s when the concentration of protein increased (Figure 5B). On the other hand, F1.0 showed no important effect on prothrombin time. It was found that the procoagulant activity of *C. urens* may be related to preferential activation of coagulation factors of the intrinsic pathway.

### 2.7. Defibrinogenating Activity

Fibrinogenolytic enzymes act by cleaving the fibrinogen molecule at sites distinct from those cleaved by thrombin, turning the fibrinogen incoagulable [20,21]. As an example, fibrinogenolytic enzymes of snake venoms, associated with activators from coagulation cascade (procoagulant toxins), can contribute to the production of a consumption coagulopathy [22]. Thus, it is important to assess whether the presence of fibrinogenolytic enzymes in F1.0 may be correlated or not with a possible blood incoagulability scenario. Therefore, we assessed the effect of F1.0 on an *in vivo* defibrinogenating activity model. Even though F1.0 presented hydrolysis of fibrinogen and procoagulant activity *in vitro*, it did not cause *in vivo* defibrinogenation when administered intraperitoneally (*i.p.*) to mice, resulting in plasma coagulability comparable to the control group (treated with 0.05 M Tris-HCl, 0.15 M NaCl pH 7.4 buffer) (data not shown). 

### 2.8. Hemorrhagic and Hemolytic Activity

Cysteine proteases present in the latex of *Calotropis gigantea*, besides presenting fibrinogenolytic activity and reduction in clotting time of the plasma also showed hemorrhagic activity, inhibited by IAA [8]. Regarding this possible toxic action, even at the higher concentrations tested, *C. urens* proteases did not induce local hemorrhage in mice *in vivo* (data not shown), suggesting that it does not act degrading the constituents of capillaries, which is supported by the absence of important hemolytic activity *in vitro* (<1% for 1000 µg of F1.0).

### 2.9. Effect of pH and Temperature on Proteolytic Activity

**Figure 6:**
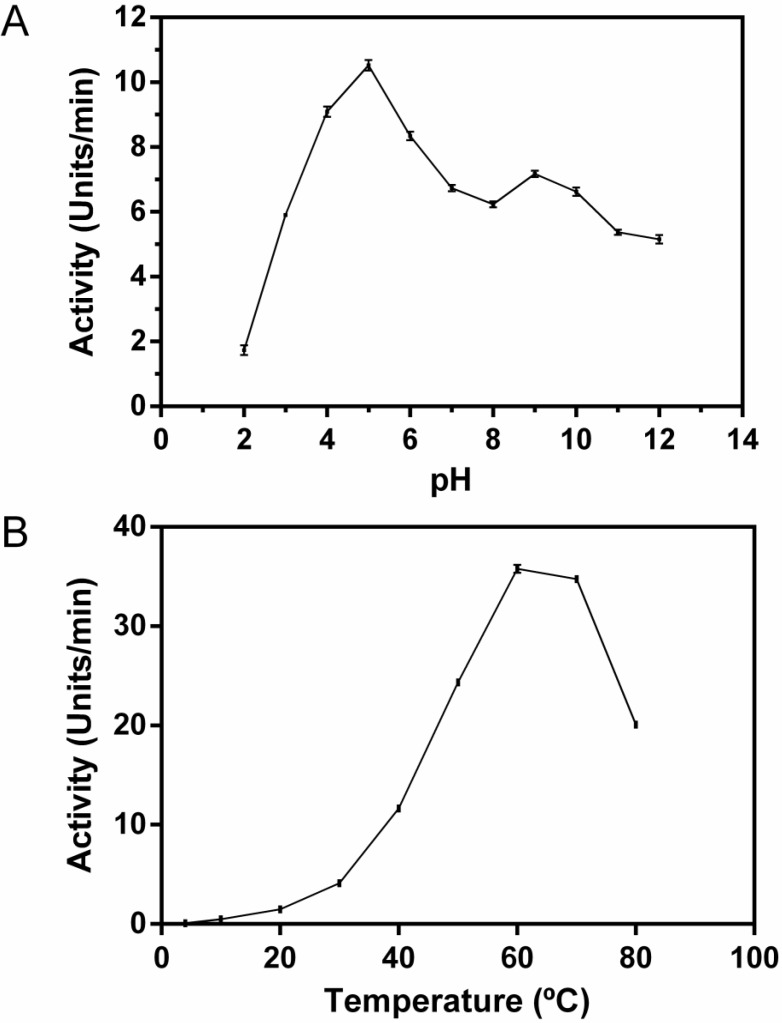
Effects of pH and temperature. (**A**) Effect of pH on the azocaseinolytic assay of fraction F1.0. The fraction F1.0 of *C. urens* (200 µg) was pre-incubated for 15 min at different pH (2.0, 3.0, 4.0, 5.0, 6.0, 7.0, 8.0, 9.0, 10.0, 11.0 and 12.0), followed by incubation with azocasein (1%) for 30 min at 37 °C in the same conditions; (**B**) Effects of temperature on the azocaseinolytic assay of fraction F1.0. The fraction F1.0 of *C. urens* (200 µg) was pre-incubated for 15 min at various temperatures (4 °C, 10 °C, 20 °C, 30 °C, 40 °C, 50 °C, 60 °C, 70 °C and 80 °C) followed by incubation with azocasein (1%) for 30 min in the same conditions. The values represent mean ± SEM (*n =* 3).

The effect of pH on the azocaseinolytic activity of F1.0 was monitored from pH 2.0–12.0. F1.0 was active at a broad pH range, especially at pH of 5.0 (*p <* 0.001) (Figure 6A). The relative activity at pH 4 and 6 were 83.6% and 80.5%, respectively. This result was similar to other cysteine proteases described in the literature, such as capparin (pH 5.0) [23] and protease of *Zingiber officinale* (pH 5.5) [24]. Evaluating the effect of temperature variation (Figure 6B), F1.0 was active at temperatures ranging from 20 °C to 80 °C, and maximum activity was observed at a temperature of 60 °C (*p <* 0.001), which is also in agreement with other cysteine proteases, such as capparin [23], protease of *Zingiber officinale* [24], procerain [25] and fibrin(ogen)olytic enzyme AMP48 [1].

### 2.10. Effect of Metal Ions, Detergents and Reducing Agents on Enzymatic Activity

The effect of some metal ions and detergents on azocaseinolytic activity was analyzed (Table 1). Metal ions are important cofactors for the catalytic activity of several enzymes. Among the major metal ions that are required for enzymatic reactions are Zn^2+^, Ca^2+^, Mg^2+^ and Mn^2+^. However, some ions can act as enzymatic inhibitors, such as Hg^2+^, which has often been associated with the inhibition of cysteine proteases [24,26]. The metal salts CoCl_2_, CuSO_4_, HgCl_2_, NiSO_4_, ZnCl_2_, significantly inhibited the enzymatic activity in *C. urens* (*p <* 0.001). The inhibition of salts upon the enzymatic activity as observed was also exhibited for other cysteine proteases isolated from plants, such as *Triticum aestivum* (inhibition in the presence of Cu^2+^, Ca^2+^, Hg^2+^, Ni^2+^ and Zn^2+^), *Zingiber officinale* (inhibition in the presence of Cu^2+^ and Hg^2+^), and *Capparis spinosa* (inhibition in presence of Hg^2+^ and Co^2+^) [23,24,27]. Inhibition of proteases by Hg^2+^is suggestive of the existence of amino acids containing group-SH at or near the active site [24].

The nonionic surfactant Triton X-100 did not affect the enzymatic activity significantly (*p >* 0.05). Tween-20, another nonionic surfactant, reduced only discreetly its activity. However, in the presence of SDS, the enzyme activity was significantly reduced (in over 60%). SDS is an anionic surfactant and has high negative charge and a hydrophobic tail that interacts with the polypeptide chains, disrupting hydrophobic interactions [28].

**Table 1:** Effects of metal ions and detergents on the azocaseinolytic activity of fraction F1.0. # Control (H_2_O) with 100.0 ± 1.7 relative activity. * *p* < 0.05, ** *p* < 0.01 and *** *p* < 0.001 compared to control (H_2_O).

Metal ion and detergents	Concentration	% Relative activity #
Ba^2+^	1 mM	81.8 ± 1.1 ***
Ca^2+^	1 mM	82.4 ± 1.3 ***
Co^2+^	1 mM	12.1 ± 3.6 ***
Cu^2+^	1 mM	2.3 ± 3.0 ***
Hg^2+^	1 mM	1.8 ± 6.0 ***
Ni^2+^	1 mM	5.1 ± 2.3 ***
Mg^2+^	1 mM	88.6 ± 3.6 **
Mn^2+^	1 mM	56.1 ± 1.1 ***
Zn^2+^	1 mM	15.5 ± 3.9 ***
Tween 20	1%	92.5 ± 1.1 *
Triton	1%	106.4 ± 2.3
SDS	1%	36.3 ± 3.9 ***

**Figure 7:**
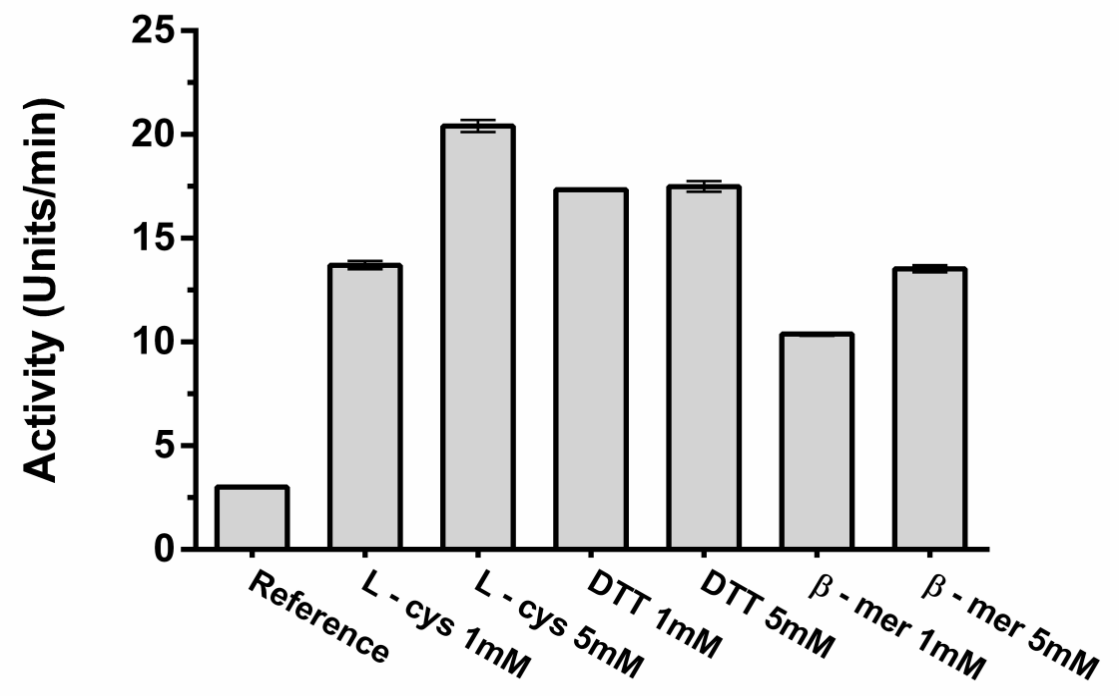
Effect of reducing agents on the azocaseinolytic activity of fraction F1.0. (l-cys) l-cysteine; (DTT) Dithiothreitol; (β-mer) β-mercaptoethanol. The values represent mean ± SEM (*n =* 3).

For enzymes belonging to the class of cysteine protease, the assessment of hydrolytic activation with thiol-specific reducing agents such as l-cysteine, β-mercaptoethanol and DTT are important. Despite these agents can act contributing to increased activity, an excess may result in reduction of disulfide bonds and thus trigger enzymatic inactivation. These tests are important and contribute to verify if the enzyme under study belongs to the class of cysteine protease [25,29]. *C. urens* had its enzymatic activity strongly increased in the presence of reducing agents (Figure 7). For instance, l-cysteine (5 mM) increased about 7 times the enzymatic activity (*p <* 0.001), contributing to the conclusion that cysteine proteases are the enzymes predominantly found in the leaves of this plant species.

## 3. Experimental

### 3.1. Materials

Azocasein, bovine fibrinogen, albumin from bovine serum, and protease inhibitors E-64 and phenylmethylsulphonyl fluoride (PMSF) were purchased from Sigma Aldrich (St Louis, MO, USA). Bicinchoninic acid (BCA) assay kit was purchased from Pierce (Rockford, IL, USA). All reagents of SDS-PAGE were purchased from GE Healthcare (Piscataway, NJ, USA). All other reagents and solvents used were of analytical grade. The water used was purified by reverse osmosis. The leaves used for characterization of biological activity in this work were collected in Ceará-Mirim city (5°36'08.34''S 35°26'48.77''O), Rio Grande do Norte, Brazil. The species studied was identified in the “Herbário Parque das Dunas”, Department of Botany and Zoology, at the Federal University of Rio Grande do Norte, where the specimen was inserted into the collection with protocol number 14,065. The human blood samples were collected from healthy volunteers.

### 3.2. Animals

Male and female Swiss mice (32 g ± 3 g) 6–8 weeks of age were obtained from the “Biotério de Criação de Animais do Centro de Ciências da Saúde da UFRN”. The animals were maintained under a controlled temperature (22 ± 2 °C) and with free access to water and commercial feed. The experiments were performed in accordance with the guidelines established for the care of laboratory animals. This study was approved by the “Research Ethics Committee on Animal Use” at the Federal University of Rio Grande do Norte, under protocol number 028/2009.

### 3.3. Plant Material and Preparation of Crude Enzyme Source

Samples of plant tissue previously washed with distilled water were pulverized in liquid nitrogen. The fine powder was weighed and added to buffer 0.05 M Tris-HCl, 0.15 M NaCl, pH 7.5 in the ratio 1:2 (w/v), under stirring at 4 °C for 2 h. After centrifugation for 30 min at 12,000 g at 4 °C, the supernatant (crude extract) was precipitated with acetone (1:2, 1:1 and 2:1, v/v, acetone: extract) at 4 °C for 4 h, to obtainment of fractions F0.5, F1.0 and F2.0, respectively. After centrifugation for 30 min at 12,000 g at 4 °C, the precipitates were collected and dried under vacuum (Concentrator Plus, Eppendorf, Hamburg, Germany) and then resuspended in the same extraction buffer and stored at −20 °C.

### 3.4. Protein Concentration Determination

Protein concentrations were analyzed using the BCA assay kit following the manufacture’s instruction. Aliquots of sample (25 μL) were added to microwell plate and mixed with BCA reagent (200 μL). The plate was incubated for 30 min at 37 °C and the absorbance measured at 562 nm with a microwell plate reader (µQuant™–BioTek, Winooski, VT, USA). Bovine serum albumin (BSA–Sigma) (0.025–2.0 mg/mL) was used as a standard calibration curve.

### 3.5. Azocaseinolytic Assay

The azocaseinolytic activity of F0.5, F1.0 and F2.0 was performed as previously described [30] with modifications, using 1% azocasein as substrate in 0.05 M Tris-HCl, pH 7.5. The reaction was performed in 350 µL assay volume containing 100 µL of 1% azocasein and 250 µL buffer 0.05 M Tris-HCl, 0.15 M NaCl, pH 7.5 with the fractions at various protein concentrations. The mixture was incubated for 30 min at 37 °C. The reaction was stopped by adding 150 µL of 20% TCA, placed on ice for 10 min and centrifuged at 10,000 g for 10 min. The supernatant was mixed with an equal volume of 2 M NaOH solution and absorbance was measured at 440 nm, using the reagent as a blank. One unit of azocaseinolytic activity was defined as the amount of enzyme required to cause an increase in absorbance (440 nm) of 0.001.

### 3.6. SDS-PAGE

The electrophoretic profile of F1.0 was determined by sodium dodecyl sulfate-polyacrylamide gel electrophoresis (SDS-PAGE) 15%, using the minigel system GE Healthcare (Piscataway, NJ, USA) [31]. The relative molecular mass of proteins was estimated by comparing the electrophoretic migration pattern of a protein mixture obtained commercially (Gibco-BRL Life Technologies, Gaithersburg, MD, USA). The gels were stained with silver staining kit (GE Healthcare).

### 3.7. Fibrinogenolytic Activity

Fibrinogen hydrolyzing activity of fraction F1.0 was determined as previously described [8] with modifications. Briefly, 50 µg of fibrinogen in 0.05 M Tris-HCl, 0.15 M NaCl, pH 7.5 were incubated with 25 µL of sample F1.0 at different protein concentrations (0.02–0.18 µg/µL) for 60 min. In time-dependent assay, fibrinogen was incubated with 0.18 µg of F1.0 proteins in a reaction volume 50 µL for different time intervals (0.5, 1, 2, 3, 4, 5, 10, 15, 20, 30, 60, 120 min). The reaction was stopped by adding 25 µL of sample buffer containing 10% β-mercaptoethanol and 2% SDS and subjected to SDS-PAGE (12%). The fibrinogen-hydrolyzing pattern was visualized by staining with Coomassie brilliant blue R-250. Alternatively, for inhibition studies, enzyme samples of F1.0 were pre-incubated with or without specific protease inhibitors (1 mM E-64, 1mM PMSF or 1 mM EDTA) for 15 min and further assay was carried out as described above. 

### 3.8. In-Gel Protease Assay (Zymography)

In-gel protease assay was performed as previously described [32] with modifications, using 0.3% gelatin, 0.3% albumin or 0.15% fibrinogen co-polymerized with the resolving gel. The sample was mixed with non-reducing sample buffer and was run on 15% PAGE. After electrophoresis, gel was washed two times with 2.5% Triton X-100 for 20 min, and rinsed with distilled water for five times to remove all traces of SDS. Then gels were immersed in developing buffer 0.05 M Tris-HCl, 0.15 M NaCl, pH 7.5 and incubated for 16 h at 37 °C for protease activity. The reaction was stopped and stained by adding 0.15% Coomassie brilliant blue R-250 in water:methanol:acetic acid (50:40:10, v/v/v) and destained with same solution without dye to visualize the clear hydrolytic zone.

### 3.9. Procoagulant Activity

Clotting activity of F1.0 was studied using citrated human plasma, as previously described [6] with modifications. Fresh human blood was mixed with 0.11 M Tri-sodium citrate in the ratio 9:1. The tubes were centrifuged for 15 min at 1000 g. The supernatant (plasma) was used for testing. To 150 µL of plasma pre warmed (37 °C), different concentrations of F1.0 proteins in 0.05 M Tris-HCl, 0.15 M NaCl, pH 7.4 were added. The clotting time was measured in CLOTimer analyzer (Drake Eletrônica e Comércio Ltda., São José do Rio Preto, Brazil), which detects the sharp variation of the optical density of the sample at the instant of clotting. This study was approved by the “Research Ethics Committee in Humans” at the Federal University of Rio Grande do Norte, under protocol number 092/2009.

### 3.10. APTT and PT Tests

APTT and PT were measured in CLOTimer analyzer, as previously described [9] with modifications. For the determination of APTT, 60 μL of citrated plasma was added 40 μL of buffer or F1.0 at different concentrations (100 μg–200 μg), homogenized and pre-incubated for 1 min (37 °C). Subsequently, 100 μL of APTT reagent (phospholipids and ellagic acid–CLOT) were added and incubated for 3 min at 37 °C. After the incubation period, coagulation was initiated by addition of 100 μL CaCl_2_ 25 mM (CLOT). For PT determination, 60 μL of citrated plasma was added to 40 μL of buffer or different concentrations fraction (100 μg–200 μg), homogenized and pre-incubated for 4 min at 37 °C. After the incubation period, 200 μL PT reagent (extract of rabbit brain and calcium chloride–CLOT) was added and the coagulation time was measured. 

### 3.11. Detection of Fibrinolytic Activity Using SDS-PAGE

The plasma clot hydrolytic activity of fraction F1.0 was studied using citrated human plasma, as previously described [7] with modifications. Treated blood was centrifuged for 15 min at 1000 g to separate plasma. Plasma (100 µL) was mixed with 30 µL of 250 mM CaCl_2_ to get a soft fibrin clot. The clot was thoroughly washed with 0.05 M Tris-HCl, 0.15 M NaCl, pH 7.5 (5 times). The washed clot was then incubated with different doses of fraction F1.0 (0.09 µg/µL–1.5 µg/µL), in a 50 µL reaction mixture at 37 °C for 60 min. The reaction was stopped by adding 25 µL sample buffer containing 2% SDS and 10% β-mercaptoethanol, boiled for 3 min and centrifuged for 10 min at 800 g. The supernatant was used to analyze the hydrolyzing pattern of plasma clot in 12% SDS-PAGE according to the method of Laemmli [31].

### 3.12. Defibrinogenating Activity

Defibrinogenant activity was tested using the method of Gene *et al.* with modifications [33]. Groups of 5 animals composed of Swiss mice (32 ± 3 g) of both sex were injected (*i.p.*) with different concentrations (1000, 500, 250 and 100 µg) of fraction of the protein extract of *C. urens* dissolved in 0.05 M Tris-HCl, 0.15 M NaCl (pH 7.4) buffer in a total volume of 200 µL. The experimental control group was given 200 µL of buffer. After 4 h, the animals were sacrificed by overdose of thiopental and blood collected by cardiac puncture. The collected material was transferred to individual microtubes and kept at ambient temperature (approximately 22 °C). After one hour, the occurrence of clot formation was visually verified.

### 3.13. Hemorrhagic Activity

The hemorrhagic activity test was performed as described in Nikai *et al.* with modifications [34]. Initially, groups of five animals (Swiss mice 32 ± 3 g) were injected into the subcutaneous dorsal region, with 100 µL of fraction F1.0 in different concentrations (1000 µg, 500 µg, 250 µg and 100 µg). After 5 h, the animals were anesthetized with thiopental and sacrificed. The dorsal skin was removed and the appearance of bleeding halos checked macroscopically and measured using a digital pachymeter (Digimess, Shiko Precision Gaging Ltd., Beijing, China). The minimum hemorrhagic dose was considered as the lowest concentration able to induce the formation of a halo with 1cm of diameter at the site of application.

### 3.14. Hemolytic Activity

Blood samples from healthy non-smoking volunteers were collected in K_3_EDTA (1.5 mg EDTA: 1 mL blood) test tubes, washed three times with saline and subjected to centrifugation at 800 g for 10 min between each washing, from which 20% red blood cell (RBC) suspension was prepared. Serial dilutions of fraction F1.0 (50–1000 µg) were added separately to each microtube containing 1 mL 0.2% RBC suspension and incubated at 37 °C for 1 h. The tubes were centrifuged for 10 min at 800 g and the degree of RBC lysis was assessed by measuring the optical density of the supernatant at 540 nm wavelength. The results were compared with negative non-treated control and a positive control (100% lysis) in which normal saline was substituted with distilled water.

### 3.15. Effect of pH and Temperature on Proteolytic Activity

The azocaseinolytic activity of F1.0 was monitored at varying pH values, to determine the optimum pH, using the specified buffers in the pH range of 2–12 as follows: for pH 2–5: 0.05 M sodium acetate buffer; pH 6–7: 0.05 M sodium phosphate buffer; pH 8–9: 0.05 M Tris-HCl; and for pH 10–12: 0.05 M glycine buffer. The optimum temperature was determined in the temperature range of 4 °C to 80 °C and the enzymatic activity of fraction F1.0 was assayed by the azocaseinolytic assay described above.

### 3.16. Effect of Metal Ions, Detergents and Reducing Agents on Enzymatic Activity

The effect of metal ions on the enzyme activity was determined using different salts in 0.05 M Tris-HCl, 0.15 M NaCl, buffer, pH 7.5. Fraction F1.0 pre-incubated with 1 mM of the salts CoCl_2_, NiSO_4_, MnCl_2_, CaCl_2_, MgCl_2_, CuSO_4_, HgCl_2_, BaCl_2_ or ZnCl_2_ (at 37 °C for 15 min) were tested and the assay was performed in triplicate using azocasein 1% as a substrate. Alternatively, fraction F1.0 was pre-incubated with 1 mM or 5 mM of the reducing agents DTT, β-mercaptoethanol or l-cysteine, or with 1% solutions of the detergents triton x-100, SDS or tween-20 in 0.05 M Tris-HCl, 0.15 M NaCl, pH 7.5 buffer, at 37 °C for 15 min. The enzyme activity was determined in triplicates using azocasein as substrate, as described above. 

### 3.17. Statistical Analysis

One-way ANOVA with Tukey’s post test was performed using GraphPad Prism version 5.00 for Windows, GraphPad Software (San Diego, CA, USA). *P-*values lower than 0.05 were considered significant.

## 4. Conclusions

The study of a protein-rich fraction of *C. urens* revealed the presence of cysteine proteases, which are active in acidic pH and high temperature, with increased activity in the presence of reducing agents. Absence of toxic effects assessed in models of hemorrhagic and hemolytic activities, and the detection fibrinogenolytic, pro-coagulant and fibrinolytic activity were verified and indicate the potential application of proteases present in *C. urens* fraction F1.0 as anti-hemorrhagic, thrombolytic and healing wound agent. Among the future prospects of this work is the purification of the enzyme(s) mainly responsible for these activities.
